# Supplementary material for: A Novel Uremic Score Reflecting Accumulation of Specific Uremic Toxins More Precisely Predicts One-Year Mortality after Hemodialysis Commencement: A Retrospective Cohort Study
Source: Toxins (Basel). 2020 Oct 1;12(10):634. doi: 10.3390/toxins12100634 (PMC7601006; doi:10.3390/toxins12100634)
Supplement: Supplementary file 1 [file toxins-12-00634-s001.pdf]

Supplementary Materials: A Novel Uremic Score Reflecting Accumulation of Specific Uremic Toxins More Precisely Predicts One-Year Mortality after Hemodialysis Commencement: A Retrospective Cohort Study

Yohei Arai, Shingo Shioji, Hiroyuki Tanaka, Daisuke Katagiri and Fumihiko Hinoshita

Table S1. Types of previous malignant diseases.

Types of Malignancies	Cases
Gastrointestinal cancer	16
Urological cancer	10
Gynecologic cancer	4
Lung cancer	3
Multiple cancer	3
Other	3

Table S2. Crude and adjusted hazard ratios for a one-point increase in adjusted uremic score using adjusted ΔcAG calculated with serum sodium level corrected for total protein concentration for the primary outcome (n = 170). Adjustment of the multivariate Cox proportional hazards model was performed for baseline covariates: Model 1, demographic data (age ≥ 75 years, sex, BMI < 20 kg/m^2) and clinical status; Model 2, Model 1 plus laboratory data (eGFR > 7 mL/min/1.73 m^2, systolic blood pressure < 110 mmHg, hemoglobin < 7 g/dL, albumin < 3 g/dL, phosphate > 6 mg/dL); Model 3, Model 2 plus comorbidities and medications. Abbreviations: ΔcAG, change in anion gap corrected for albumin; HR, hazard ratio; CI, confidence interval; p-value, two-sided probability value; BMI, body mass index; eGFR, estimated glomerular filtration rate.

Cox Proportional Hazard Model	HR (95% CI)	*p*-value
One point increase in the adjusted uremic score		
Unadjusted model	1.68 (0.94, 3.00)	0.076
Model 1	1.40 (0.73, 2.65)	0.301
Model 2	2.22 (0.95, 5.18)	0.064
Model 3	6.76 (1.27, 35.9)	0.025

Table S3. Crude hazard ratios for the primary outcome (n = 230). Abbreviations: HR, hazard ratio; CI, confidence interval; p-value, two-sided probability value; BMI, body mass index; eGFR, estimated glomerular filtration rate; BUN, blood urea nitrogen; ΔcAG, change in anion gap corrected for albumin; β2MG, β2-microglobulin; RAS inhibitor, renin-angiotensin system inhibitor; ESA, erythropoiesis stimulating agent.

Cox Proportional Hazard Model	Univariate Analysis	
	HR (95% CI)	*p*-value
Demography		
Age	1.04 (1.00–1.08)	0.049
Male gender	0.29 (0.11–0.78)	0.015
BMI	0.85 (0.74–0.98)	0.035
Clinical status		
Presence of hemodialysis shunt	0.19 (0.07–0.51)	0.001

Nephrology care (>6 months)	0.43 (0.15–1.25)	0.124
Laboratory data		
eGFR	1.32 (1.08–1.60)	0.005
Systolic blood pressure	0.95 (0.92–0.97)	<0.001
Hemoglobin	0.89 (0.63–1.26)	0.525
Albumin	0.34 (0.16–0.70)	0.003
Phosphate	1.06 (0.79–1.43)	0.669
BUN	1.02 (1.01–1.04)	<0.001
ΔcAG	1.08 (0.96–1.22)	0.172
β2MG	1.01 (0.92–1.10)	0.787
Comorbidities		
Diabetes	0.39 (0.14–1.08)	0.072
Cardiovascular disease	1.42 (0.53–3.81)	0.484
Malignant disease	2.28 (0.79–6.57)	0.125
Medications		
RAS inhibitor	1.26 (0.45–3.47)	0.651
Statin	1.21 (0.45–3.27)	0.694
ESA	0.09 (0.03–0.26)	<0.001
